# Multiple metabolic comorbidities and their consequences among patients with peripheral arterial disease

**DOI:** 10.1371/journal.pone.0268201

**Published:** 2022-05-10

**Authors:** Young Shin Park, Gi Wook Ryu, Mona Choi

**Affiliations:** 1 Mo-Im Kim Nursing Research Institute, Yonsei University College of Nursing, Seoul, Republic of Korea; 2 College of Nursing and Brain Korea 21 FOUR Project, Yonsei University College of Nursing, Seoul, Republic of Korea; 3 Department of Nursing, Hansei University, Gyeonggi-do, Republic of Korea; Ehime University Graduate School of Medicine, JAPAN

## Abstract

Older adults with peripheral arterial disease (PAD) have increased risks of major cardiovascular events and hospitalization. Metabolic comorbidities, such as hypertension, diabetes mellitus, and dyslipidemia, are common risk factors for these unfavorable health events. This study aimed to determine how multiple metabolic comorbidities affect the risk of adverse health outcomes in older adults with PAD. A retrospective cohort study was adopted using administrative claim data from the Health Insurance Review and Assessment Service Agency. The study sample included 3,122 older adults (≥ 65 years old) with at least one PAD diagnosis in an inpatient setting from 2011 to 2013 and who had at least one follow-up visit after hospitalization by 2018. The three metabolic comorbidities were defined as having at least two diagnostic claims or one prescription per year of anti-hypertensive, anti-diabetic, or anti-dyslipidemic medications for the corresponding diagnosis. The outcome variables included major adverse cardiovascular events (MACEs) and major adverse limb events (MALE). Kaplan-Meier survival curves demonstrated that PAD patients with more metabolic comorbidities had shorter MACE-free and MALE-free periods. Adjusted Cox proportional hazards analyses suggested significant additive effects of multiple metabolic comorbidities on increased risks of MACE and MALE among older adults with PAD. Lower socioeconomic status and non-metabolic comorbidities also increased the risk of MACE. Male sex, being young-old (65–74 years), and a higher proportion of inpatient claims increased the risk of MALE. The findings of this study suggest the need for a comprehensive care program for older adults with PAD and metabolic comorbidities. In addition, the healthcare utilization pattern should be considered when designing preventive care for older patients with comorbidities to manage PAD at an earlier stage.

## Introduction

Globally, more than 200 million people suffered from peripheral arterial disease (PAD) in 2010, with an increase in PAD incidence of 23.5% in the past ten years [1]. In a study using the US healthcare administrative database from 2003 to 2008, PAD had a mean annual prevalence of 10.7% and a mean annual incidence of 2.4% [2]. The prevalence of PAD ranged from 3% to 4% in middle-aged adults and 13% to 14% in older adults [3]. The global disease burden from death and disability due to PAD has also increased over the 20 years from 1990 to 2010 [4]. PAD is also the third leading cause of atherosclerotic vascular disease [1]. The economic costs associated with hospitalization due to PAD are higher than those of coronary artery disease or cerebrovascular disease, which need vascular interventions as major cardiovascular events [5]. People with PAD suffer from functional limitations related to symptoms of claudication or limb loss, which result in a decrease in quality of life [6].

A previous study revealed higher rates of cardiovascular death events and the combined outcomes of major cardiovascular events (MACE), major adverse limb events (MALE), and hospitalization in PAD patients than in those people having coronary artery diseases [7]. Metabolic diseases are highly associated with PAD [8]. PAD patients have a higher risk of hypertension, diabetes mellitus, and lipid disorders than those without PAD [9, 10]. These comorbid metabolic diseases are also associated with increased risks of major cardiovascular events including myocardial infarction (MI), limb ischemia, or stroke as they share common causes or risk factors [11–13]. For example, diabetes concomitant with PAD is known to increase the risks of cardiovascular diseases. However, the magnitude of the risks attributed to diabetes differs based on other comorbidities present [14]. Previous studies have examined the negative impact of having a metabolic disease, either diabetes or vascular disease, in the setting of PAD. However, studies have not examined the pattern of coexisting metabolic diseases, such as hypertension, diabetes, and dyslipidemia, and their effects on PAD-related adverse health outcomes.

As these three metabolic diseases commonly coexist with PAD, it is important to focus on the influence of multiple metabolic comorbidities on the older and more vulnerable populations suffering from PAD. However, this pattern is less understood in older Korean populations. Thus, this study examined the association between multiple metabolic comorbidities, such as hypertension, diabetes, and dyslipidemia, and the risks of MACE and MALE among Korean older adults with PAD.

## Materials and methods

### Ethical approval and study design

This study was approved by the Yonsei University Institutional Review Board (Y-2019-0105). This study performed secondary data analysis through an observational and retrospective cohort study design, which analyzed patient-level individual data linking administrative claim data from the Health Insurance Review and Assessment Service Agency (HIRA) of South Korea.

### Data source and study population

**Fig 1:**
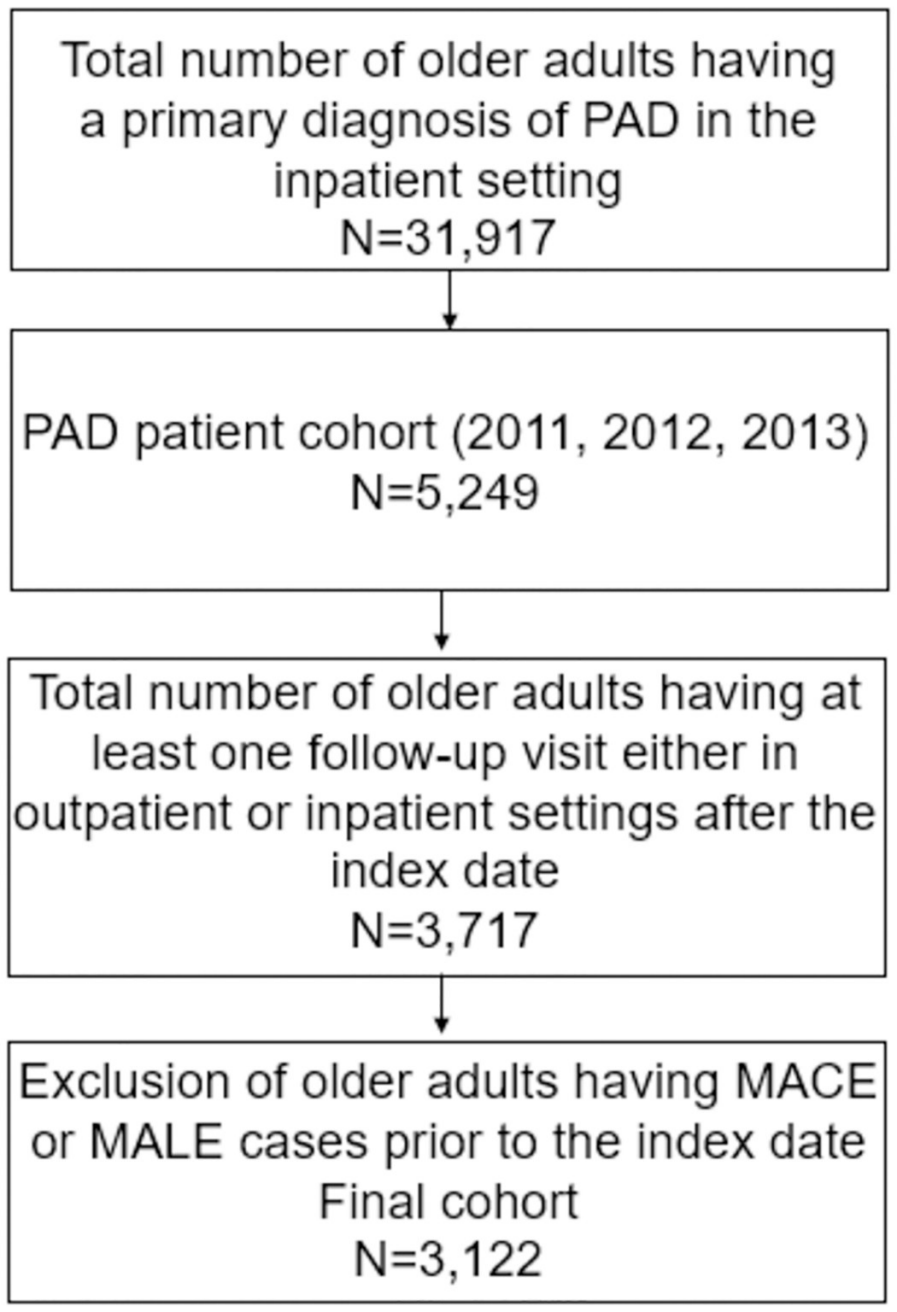
Flow diagram of the cohort selection process.

Korea adopts the single-payer system and the government-administered universal insurance healthcare system. The datasets used for this study were obtained from the HIRA administrative claim data. The study sample included PAD patients aged ≥ 65 years separated into three age groups (young-old: 65–74 years; old-old: 75–84 years; oldest-old: ≥85 years) with at least one claim of PAD diagnosis as a primary diagnosis in inpatient visits from January 2011 to December 2013 and had at least one follow-up outpatient or inpatient visit with any PAD diagnosis within one year after the index hospitalization [15]. An incident PAD patient was defined as an individual hospitalized with a primary diagnosis of PAD during the study period using the International Classification of Diseases, Tenth Revision (ICD-10) (I70.2, I73.8, I73.9, I74.3, I74.4, I74.5, I74.8, and I74.9). Patients with a PAD diagnosis 2 years before (2009–2010) the study period were excluded to include incident cases. The earliest date of PAD diagnosis, on or after January 2011, was defined as the baseline index date. We eliminated all cases with claims corresponding to either MACE or MALE before the study period. Finally, a total of 3,122 older adults with PAD were included for analysis (Fig 1).

### Independent variables

Metabolic comorbidities were defined as having at least two claims per year of hypertension (HTN), type 1 or type 2 diabetes mellitus (DM), and dyslipidemia with ICD-10 diagnosis codes (HTN: I10, I11, I12, I13, I15; DM: E10, E11, E12, E13, E14; dyslipidemia: E78), or one claim with the prescription of anti-hypertensive, anti-diabetic, or anti-dyslipidemic medication per year under these diagnosis codes [15]. The details of ICD-10 codes for metabolic comorbidities are provided in the S1 Table.

This study aimed to quantitatively examine how metabolic comorbidities affect adverse health outcomes. We determined the number of participants in the cross tables with HTN or DM, HTN or dyslipidemia, and DM or dyslipidemia. Approximately 30% of the participants (n = 952) had hypertension and dyslipidemia, making this the highest among the other overlapping patterns of paired metabolic diseases. Descriptive cross tables of the number and percentage of participants with HTN or DM, HTN or dyslipidemia, and DM or dyslipidemia are presented in S2 Table. Finally, the total number of metabolic comorbidities of hypertension, diabetes, or dyslipidemia was used as an independent variable to measure the adverse health outcomes.

### Outcome variables

This study considered adverse health consequences that took place after the index date. These included major adverse cardiovascular events (MACEs), such as including myocardial infarction, ischemic stroke, and unstable or stable angina, and major adverse limb events (MALEs), such as major amputation (amputation of the extremities of the pelvis, thigh, upper arm/forearm/lower leg), minor amputation (hand/foot, finger/toe), revascularization, and open surgery [16–18]. The end of the follow-up period for this study was December 31, 2018 (censored). The details of ICD-10 codes for MACE are provided in the S3 Table and the HIRA procedures codes for MALE are provided in the S4 Table [18].

### Covariate variables

Covariates including the age, sex, type of insurance (insured and others including medical aid and veterans), and the proportion of days covered (PDC), were calculated 3 months after the index date. An 80% PDC indicated good compliance [19–21]. The PDCs of anti-hypertensive, anti-diabetic, anti-dyslipidemic, and antiplatelet medications were included as binary variables. The details of medications are provided in the S5 Table. The total number of other comorbid diseases included in this study were the following: congestive heart failure, dementia, chronic pulmonary diseases, rheumatologic diseases, peptic ulcer diseases, mild, moderate or severe liver diseases, hemiplegia or paraplegia, renal disease, and malignancy. Finally, the proportion of inpatient claims to the total number of outpatient and inpatient claims was calculated to adjust for the severity of diseases.

### Data analysis

Descriptive statistics of the participants at the index date were calculated. Kaplan–Meier survival curves were used to compare the overall MACE-free and MALE-free among PAD patients with a different number of metabolic comorbidities. Cox proportional hazards models examined the association between the number of metabolic comorbidities and MACE-free or MALE-free periods. We examined the proportional hazard assumption using plots of the log(-log) survival function. Models were adjusted for the covariates (age, sex, type of insurance, PDC of anti-hypertensive, anti-diabetic, anti-dyslipidemic, and antiplatelet medications), the total number of comorbid diseases, and the proportion of inpatient claims to the total number of claims per patient.

## Results

A descriptive analysis of the participants’ characteristics at the index date is shown in Table 1. Approximately 50% of the participants were young-old (65–74 years), and most were men (70%) and insured (89%). PAD patients with hypertension comprised 62% of the sample; 32% were diabetic, and 40% had dyslipidemia. More than half of the population were taking anti-hypertensive medications prescribed as of the index date. Calcium channel blockers (29.50%) were most frequently prescribed, followed by angiotensin II receptor blockers (ARBs) (18.29%), diuretics (18.03%), and alpha or beta-blockers (17.75%). Approximately 17% of the participants were prescribed anti-diabetic medications. Around 30% of the older patients with PAD were given anti-dyslipidemic medication, with statins as the major prescribed drug (30%). Most patients (80.59%) were prescribed antiplatelet medications and 60% of these participants were given aspirin. The mean number of comorbidities was 0.74 (±0.88) and the mean proportion of inpatient claims to the total number of claims was 0.22 (±0.26).

**Table 1:** Participants characteristics at index date (n = 3,122).

	n (%)
Age	
65~74	1,551 (49.68)
75~84	1,249 (40.01)
85+	322 (10.31)
Sex	
Male	2,135 (68.39)
Female	987 (31.61)
Insurance type	
Insured	2,768 (88.66)
Others^a^	354 (11.34)
Metabolic disease	
Hypertension	1,930 (61.82)
Diabetes Mellitus	1,000 (32.03)
Dyslipidemia	1,262 (40.42)
Anti-hypertensive medication	1,618 (51.83)
Diuretic	563 (18.03)
ARB	571 (18.29)
ACEI	194 (6.21)
Alpha-Beta blocker	554 (17.75)
CCB	921 (29.50)
Other	217 (6.95)
Anti-diabetic medication	542 (17.36)
Anti-dyslipidemic medication	913 (29.24)
Statin	872 (27.93)
Antiplatelet medication	2,516 (80.59)
Aspirin	1,822 (58.36)
Clopidogrel	1,314 (42.09)
	Mean±SD
Number of comorbidities	0.74 (0.88)
Proportion of inpatient claim	0.22 (0.26)

ARB, angiotensin II receptor blocker; ACEI, angiotensin-converting enzyme inhibitor; CCB, calcium channel blocker; SD, standard deviation.

^a^Other types of insurance included medical aid and veterans.

Less than a quarter of the total participants (24.12%) had no metabolic comorbidities (Table 2). Approximately 30% of the sample had one or two metabolic comorbidities with PAD. Finally, 13% of the participants had all three metabolic comorbidities at the same time.

**Table 2:** Number of participants by the total number of metabolic comorbidities with PAD (n = 3,122).

Group	n (%)
1 (No metabolic comorbidity)	753 (24.12)
2 (One metabolic comorbidity)^a^	965 (30.91)
3 (Two metabolic comorbidities)^b^	985 (31.55)
4 (Three metabolic comorbidities)^c^	419 (13.42)

^a^One metabolic comorbidity means having one metabolic disease among hypertension, diabetes, or dyslipidemia with PAD.

^b^Two metabolic comorbidities mean having two metabolic diseases among hypertension-diabetes, hypertension-dyslipidemia, or diabetes-dyslipidemia at the same time.

^c^Three metabolic comorbidities mean having all metabolic diseases including hypertension, diabetes, and dyslipidemia.

One-third of the participants (33.73%) experienced MACEs. Approximately 21% had angina pectoris, 10% had an ischemic stroke, and less than 4% had a myocardial infarction during the study period (Table 3). MALE occurred in 17% of the patients, with revascularization (12.01%) as the most frequent procedure. Open surgeries (3.01%), major amputations (1.15%), and minor amputations (2.24%) were rarely performed.

**Table 3:** MACE or MALE occurrence during the study period (n = 3,122).

MACE	n (%)	MALE	n (%)
Total case	1,053 (33.73)	Total case	518 (16.59)
Myocardial infarction	116 (3.72)	Revascularization	375 (12.01)
Angina pectoris	655 (20.98)	Open surgery	94 (3.01)
Ischemic stroke	328 (10.51)	Major amputation	36 (1.15)
		Minor amputation	70 (2.24)

MACE, major adverse cardiovascular events; MALE, major adverse limb events.

**Fig 2:**
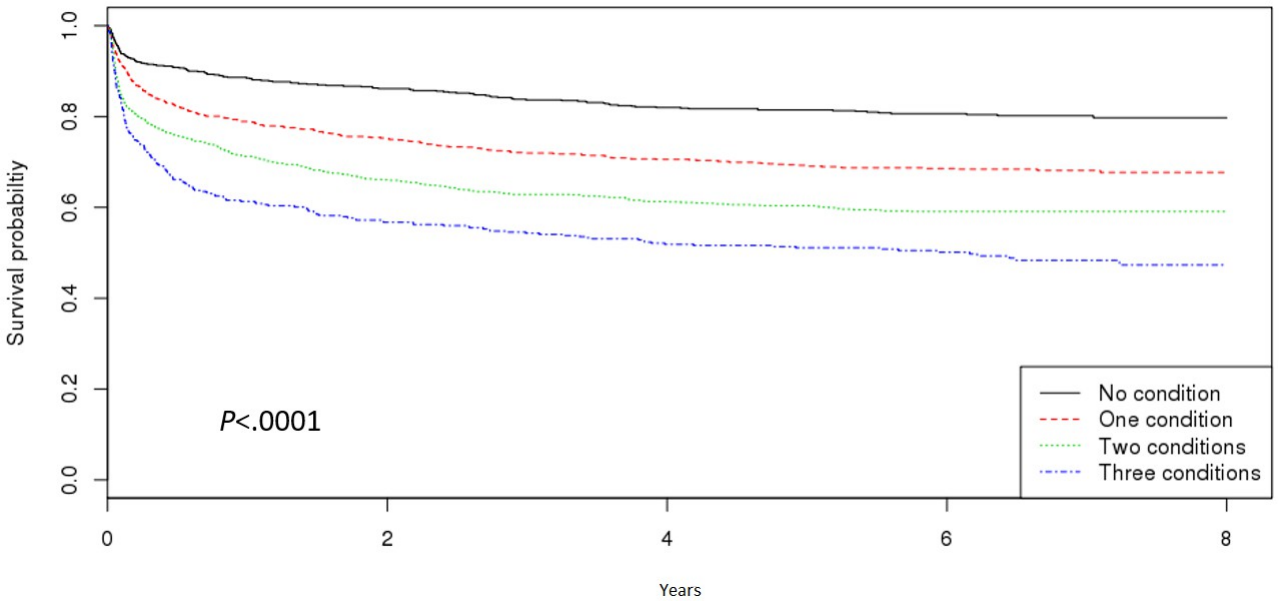
Survival curves by the number of metabolic comorbidities on MACE.

Kaplan-Meier survival curves demonstrated that PAD patients with more metabolic comorbidities had a shorter MACE-free period (Fig 2). The risks of MACE associated with the number of metabolic comorbidities were assessed in 3,122 old PAD patients (Table 4). Adjusted Cox proportional hazards analyses showed additive effects of multiple metabolic comorbidities on MACE. Old PAD patients with metabolic comorbidities had a greater risk of MACE than those without metabolic comorbidity. The adjusted hazard ratio (aHR) of the risk of MACE from having one metabolic comorbidity was 1.69 (95% confidence interval [CI]: 1.38–2.06; p < 0.0001). Having two metabolic comorbidities further increased the risk of MACE (aHR: 2.30; 95% CI: 1.90–2.80; p < 0.0001) in older adults with PAD. The presence of all three metabolic comorbidities most significantly increased the risk of MACE (aHR: 3.06; 95% CI: 2.45–3.82; p < 0.0001). Participants under medical aid and veteran insurance were at greater risk of MACE than insured old PAD patients (aHR: 1.24; 95% CI: 1.03–1.49; p = 0.0231). Lastly, the presence of other comorbidities subsequently heightened the risk of MACE to an aHR of 1.12 (95% CI: 1.04–1.20; p = 0.0013).

**Table 4:** Association of MACE with number of metabolic comorbidity groups (n = 3,122).

	Hazard ratio	95% CI	P*
**Number of metabolic comorbidities**			
**One (vs. none)**	**1.69**	**(1.38, 2.06)**	**< .0001**
**Two (vs. none)**	**2.30**	**(1.90, 2.80)**	**< .0001**
**Three (vs. none)**	**3.06**	**(2.45, 3.82)**	**< .0001**
**Male (vs. Female)**	**1.16**	**(1.01, 1.34)**	**0.0331**
Age			
75–84 (vs. 65–74)	1.01	(0.89, 1.15)	0.8840
85+ (vs. 65–74)	0.79	(0.62, 1.01)	0.0567
Anti-hypertensive medication			
Diuretic	0.85	(0.56, 1.30)	0.4554
ACEI	1.81	(0.66, 4.99)	0.2491
ARB	0.73	(0.46, 1.17)	0.1928
Alpha & Beta blocker	0.63	(0.38, 1.05)	0.0738
CCB	0.82	(0.54, 1.25)	0.3534
Other	0.74	(0.47, 1.15)	0.1818
Anti-diabetic medication	1.01	(0.73, 1.41)	0.9473
Anti-dyslipidemic medication	1.37	(0.95, 1.97)	0.0909
Antiplatelet medication	0.93	(0.80, 1.09)	0.3739
**Others**^a^ **(vs. insured)**	**1.24**	**(1.03, 1.49)**	**0.0231**
**Number of comorbidities**	**1.12**	**(1.04, 1.20)**	**0.0013**
%Inpatient setting	0.86	(0.65, 1.12)	0.2596

MACE, major adverse cardiovascular events; CI, confidence interval; ACEI, angiotensin-converting enzyme inhibitor; ARB, angiotensin II receptor blocker; CCB, calcium channel blocker.

Bold indicates significance at p < 0.05.

^a^Other insurance types included medical aid and veterans.

**Fig 3:**
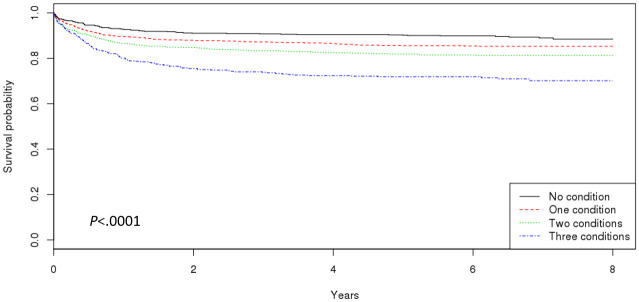
Survival curves by the number of metabolic comorbidities on MALE.

Kaplan-Meier survival curves showed that PAD patients with more metabolic comorbidities had shorter MALE-free periods (Fig 3). Adjusted Cox proportional hazards analyses showed the additive effects of multiple metabolic comorbidities on MALEs. Old PAD patients with metabolic comorbidities had a greater risk of having MALE than those without metabolic comorbidity (Table 5). Participants with one metabolic comorbidity had an aHR of 1.47 (95% CI 0.11–1.95; p < 0.0067) for MALE. Having two metabolic comorbidities with PAD also increased the risk of MALE compared to those without metabolic comorbidities (aHR: 1.98; 95% CI 1.51–2.59; p < 0.0001). When all metabolic comorbidities were present together, there was also a significant increase in the risk of MALE (aHR: 3.22; 95% CI 2.39–4.35; p < 0.0001). Male PAD patients were at a greater risk of MALE than female PAD patients (aHR: 1.64; 95% CI 1.32–2.03; p < 0.0001). Young-old PAD patients aged 65–74 years (aHR: 0.79; 95% CI 0.66–0.96; p = 0.0150) and old-old PAD patients aged 75–84 years (aHR: 0.53; 95% CI 0.36–0.77; p = 0.0010) had lower risks of MALE than the oldest-old PAD patients aged 85 years and above. Lastly, the proportion of inpatient claims to the total number of claims increased the risk of MALE with an aHR of 2.57 (95% CI 1.85–3.58; p < 0.0001).

**Table 5:** Association of MALE with number of metabolic comorbidity groups (n = 3,122).

	Hazard ratio	95% CI	P*
**Number of metabolic comorbidities**			
**One (vs. none)**	**1.47**	**(0.11, 1.95)**	**0.0067**
**Two (vs. none)**	**1.98**	**(1.51, 2.59)**	**< .0001**
**Three (vs. none)**	**3.22**	**(2.39, 4.35)**	**< .0001**
**Male (vs. Female)**	**1.64**	**(1.32, 2.03)**	**< .0001**
**Age**			
**75–84 (vs. 65–74)**	**0.79**	**(0.66, 0.96)**	**0.0150**
**85+ (vs. 65–74)**	**0.53**	**(0.36, 0.77)**	**0.0010**
Anti-hypertensive medication			
Diuretic	0.76	(0.42, 1.38)	0.3684
ACE	2.98	(0.41, 21.83)	0.2816
ARB	1.18	(0.51, 2.72)	0.7071
Alpha & beta blocker	0.92	(0.41, 2.06)	0.8389
CCB	1.10	(0.53, 2.31)	0.7982
Other	1.27	(0.58, 2.75)	0.5521
Anti-diabetic medication	1.30	(0.78, 2.18)	0.3179
Anti-dyslipidemic medication	1.45	(0.81, 2.59)	0.2169
Antiplatelet medication	0.85	(0.69, 1.05)	0.1298
Others^a^ (vs. insured)	0.97	(0.73, 1.29)	0.8462
Number of comorbidities	0.96	(0.87, 1.07)	0.4488
**%Inpatient setting**	**2.57**	**(1.85, 3.58)**	**< .0001**

MALE, major adverse limb events; CI, confidence interval; ACEI, angiotensin-converting enzyme inhibitor; ARB, angiotensin II receptor blocker; CCB, calcium channel blocker.

Bold indicates significance at p < 0.05.

^a^Other insurance types included medical aid and veterans.

## Discussion

Multiple metabolic comorbidities, such as hypertension, diabetes, or dyslipidemia had an additive effect in increasing the risk of MACE and MALE compared to older PAD patients without metabolic comorbidities. Low socioeconomic status and other comorbidities increased the risk of MACE. Meanwhile, being a male, young-old (65 to 74 years) compared to old-old and oldest-old (75 years and older), and having a higher proportion of inpatient claims out of the total claims increased the risk of MALE.

The risks of MACE or MALE doubled when two metabolic comorbidities were present and tripled when three were present. The additive impacts of metabolic comorbidities on PAD-related adverse outcomes are not supported by prior literature since related studies have not been conducted yet. However, the findings of this study are supported by evidence that hypertension, diabetes, or dyslipidemia aggravates the conditions of older adults with PAD, leading to adverse health outcomes [22, 23]. These results emphasize the need to develop a comprehensive approach for managing underlying metabolic comorbidities in patients suffering from PAD. Our analysis also encourages healthcare providers to implement further interventions for modifiable metabolic risk factors (e.g., high levels of blood pressure, cholesterol, or blood sugar) as preventable measures for adverse MACE and MALE in older PAD patients [24].

The finding that low socioeconomic status was associated with increased risk of MACE in PAD patients was consistent with prior studies. Disadvantaged individuals, such as non-white or low-income patients (e.g., Medicaid recipients) have been reported to experience more metabolic comorbidities that aggravated PAD than the socioeconomically advantaged population [25]. The development of cardiovascular events is also known to be related to some social determinants of health (e.g., income). Low income can be a barrier to accessing healthcare services, leading to chronic metabolic conditions, which are not properly managed [26].

Other diseases coexisting with PAD can also aggravate adverse cardiovascular health problems. The diseases included in the Charlson comorbidity calculation often have chronic characteristics that may interact with existing PAD and are negatively related to the development of major cardiovascular incidents. This finding was supported by a previous study that revealed a significant association between the Charlson comorbidity index score and the readmission of PAD patients [27]. In addition, the individual impact of comorbid diseases (e.g., renal disease) has been studied, and the results suggested that comorbidities play negative roles in the development of MACE in patients [28].

PAD is traditionally known as a male disease. The result that older male adults with PAD had higher risks of MALE than older female adults with PAD was consistent with previous evidence [29]. Women having better amputation-free survival periods than men across all age groups is probably due to socially constructed unhealthy behaviors in males, such as prevalent smoking, which may hasten the progress of PAD [30]. In addition, differences in health-seeking behaviors or healthcare utilization patterns may exist. Older male adults tend not to pursue treatment to manage their conditions, resulting in hospitalizations for revascularization or open surgeries more often than older female adults.

The lower risk of developing MALE in the old-old or oldest-old age groups compared to the young-old age group may reflect the difference in their healthcare utilization patterns. For old-old or oldest-old adults, it could be a reasonable decision to proceed with angiography or procedures considered only when they are experiencing severe symptomatic disabilities [31]. Compared to those age groups, young-old adults with PAD may be more proactive in pursuing revascularization procedures or open surgeries to prolong and improve the quality of life. In a prior study, the risk of mortality and cardiovascular complications was increased among PAD patients aged 80 years and over who underwent lower extremity arterial reconstruction surgery. This may be the case because the risks are higher than the benefits for patients with PAD who are 75 years old and above when they receive medical procedures or surgeries [32]. To adopt an approach that considers patient safety and treatment efficacy, many practitioners may advocate the patients’ stances over aggressive endovascular evaluation. A prior study also supports this finding on the large reduction in the number of older PAD adults (≥ 75 years) with diabetes but the main reason for the decline in this age group remains unknown [33, 34].

Lastly, the higher proportion of inpatient claims out of the total claims for PAD showed that amputation, revascularization, and open surgeries were associated with the severity of PAD. Repetitive revascularization was also conducted in older PAD patients. It is possible that some young-old patients cannot manage the symptoms of PAD, and receive repetitive procedures later in life. A previous study also found that the mortality rate was higher for hospitalized PAD patients than for PAD patients having outpatient visits [35]. This reflects the higher risk profiles of PAD patients in inpatient compared to outpatient settings. Thus, the early identification of PAD in outpatient settings will be beneficial in reducing the adverse consequences of repetitive revascularization or open surgeries.

This study is the first attempt to investigate the relationships between multiple metabolic comorbidities that frequently occur with PAD and MACE or MALE. It is also the first to use the national claim data of PAD patients in South Korea. The cohort longitudinal study design allowed for follow-ups from 5 to 8 years for MACE or MALE to explain the influences of multiple metabolic comorbidities. This study also focused on older adults aged 65 and over, who are more vulnerable to diseases like PAD. Lastly, the selection of the study cohort was based on prior studies using administrative data that assured the representativeness of older Korean adults with PAD.

This observational cohort study may be subject to potential confounding factors. We used a Cox proportional hazard model to minimize risks from those confounding factors by adjusting the covariates of demographic factors, medication prescribed, and severity of cases. With these adjustments, this secondary data analysis study inherently cannot account for unmeasured confounding variables, such as smoking behaviors or outcomes of this study. We recognize that these secondary data may not be able to measure patient adherence to medications with 100% certainty for metabolic diseases because this dataset was originally constituted to extract all PAD-diagnosed claims in the national database. This may not reflect the whole case profiles for the metabolic comorbidities. Lastly, administrative claim data could not include PAD patients possibly diagnosed as PAD patients by clinical diagnostic tests, such as the ankle-brachial index (ABI) or self-reporting limb symptoms.

For future studies, a prospective cohort study will be needed to examine the mechanism of multiple metabolic comorbidities over time in PAD-related outcomes of MACE or MALE. Future studies may also investigate the effects of comprehensive interventions for metabolic diseases with PAD to prevent MACE or MALE. With a larger sample size, there may be a need to examine the impact of multiple metabolic comorbidities on specific subgroups of PAD patients and either MACE or MALE to provide evidence for the customized intervention. Lastly, the difference in increased risk for MALE between sexes suggests that future studies may consider a sex-specific approach to examine the effects of multiple metabolic comorbidities on MALE.

## Conclusions

Multiple metabolic comorbidities, particularly hypertension, diabetes, and dyslipidemia additively increased the risks of MACE or MALE in older adults. Besides coexisting diseases with PAD, sociodemographic risk factors also increased the risks of adverse outcomes. The findings of this study suggest the need for a comprehensive treatment plan for metabolic comorbidities in older adults with PAD. In addition, healthcare utilization patterns should be considered in designing preventive care programs for older PAD patients with metabolic comorbidities to detect those in their early stages and to provide optimal care for these patients.

## Supporting information

S1 Table International Classification of Disease, 10^th^ revision (ICD-10) codes for metabolic commodities. (DOCX)

S2 Table A. Number of participants having hypertension or diabetes. B. Number of participants having hypertension or dyslipidemia. C. Number of participants having diabetes or dyslipidemia.
(DOCX)

S3 Table International Classification of Disease, 10^th^ revision (ICD-10) codes for MACE. (DOCX)

S4 Table Health Insurance Review and Assessment service (HIRA) procedures codes. (DOCX)

S5 Table A. Anti-hypertensive medications. B. Anti-diabetic medications. C. Anti-dyslipidemic medications. D. Antiplatelet medications.
(DOCX)
