# Supplementary material for: Multiple metabolic comorbidities and their consequences among patients with peripheral arterial disease
Source: PLoS One. 2022 May 10;17(5):e0268201. doi: 10.1371/journal.pone.0268201 (PMC9089858; doi:10.1371/journal.pone.0268201)
Supplement: S1 Table — (DOCX) [file pone.0268201.s001.docx]

S1 Table. International Classification of Disease, 10^th^ revision (ICD-10) codes for metabolic commodities

| Metabolic comorbidities | ICD-10 |  |
| --- | --- | --- |
| Hypertension | I10 | Essential (primary) hypertension |
|  | I11 | Hypertensive heart disease |
|  | I12 | Hypertensive renal disease |
|  | I13 | Hypertensive heart and renal disease |
|  | I15 | Secondary hypertension |
| Dyslipidemia | E78 | Disorders of lipoprotein metabolism and other lipidaemias |
| Diabetes Mellitus | E10 | Insulin-dependent diabetes mellitus |
|  | E11 | Non- Insulin-dependent diabetes mellitus |
|  | E13 | Other specified diabetes mellitus |
|  | E14 | Unspecified diabetes mellitus |
