# Supplementary material for: Multiple metabolic comorbidities and their consequences among patients with peripheral arterial disease
Source: PLoS One. 2022 May 10;17(5):e0268201. doi: 10.1371/journal.pone.0268201 (PMC9089858; doi:10.1371/journal.pone.0268201)
Supplement: S2 Table — A. Number of participants having hypertension or diabetes. B. Number of participants having hypertension or dyslipidemia. C. Number of participants having diabetes or dyslipidemia. (DOCX) [file pone.0268201.s002.docx]

S2A Table. Number of participants having hypertension or diabetes

|  | HTN (Yes) | HTN (No) |
| --- | --- | --- |
| DM (Yes) | 774 | 226 |
| DM (No) | 1,156 | 966 |

Note. DM, diabetes mellitus; HTN, hypertension.

S2B Table. Number of participants having hypertension or dyslipidemia

|  | HTN (Yes) | HTN (No) |
| --- | --- | --- |
| Dyslipidemia (Yes) | 952 | 310 |
| Dyslipidemia (No) | 978 | 882 |

Note. HTN, hypertension.

S2C Table. Number of participants having diabetes or dyslipidemia

|  | DM (Yes) | DM (No) |
| --- | --- | --- |
| Dyslipidemia (Yes) | 516 | 746 |
| Dyslipidemia (No) | 484 | 1376 |

Note. DM, diabetes mellitus.
