# Supplementary material for: Multiple metabolic comorbidities and their consequences among patients with peripheral arterial disease
Source: PLoS One. 2022 May 10;17(5):e0268201. doi: 10.1371/journal.pone.0268201 (PMC9089858; doi:10.1371/journal.pone.0268201)
Supplement: S3 Table — (DOCX) [file pone.0268201.s003.docx]

S3 Table. International Classification of Disease, 10^th^ revision (ICD-10) codes for MACE

| MACE: major cardiovascular disease | ICD-10 |  |
| --- | --- | --- |
| Myocardial Infarction | I21.0 | Acute transmural myocardial infarction of anterior wall |
|  | I21.1 | Acute transmural myocardial infarction of inferior wall |
|  | I21.2 | Acute transmural myocardial infarction of other sites |
|  | I21.3 | Acute transmural myocardial infarction of unspecified site |
|  | I21.4 | Acute subendocardial myocardial infarction |
|  | I21.9 | Acute myocardial infarction, unspecified |
|  | I22.0 | Subsequent myocardial infarction of anterior wall |
|  | I22.1 | Subsequent myocardial infarction of inferior wall |
|  | I22.8 | Subsequent myocardial infarction of other sites |
|  | I22.9 | Subsequent myocardial infarction of unspecified site |
|  | I23.0 | Haemopericardium as current complication following acute myocardial infarction |
|  | I23.1 | Atrial septal defect as current complication following acute myocardial infarction |
|  | I23.2 | Ventricular septal defect as current complication following acute myocardial infarction |
|  | I23.4 | Rupture of chordae tendineae as current complication following acute myocardial infarction |
|  | I23.5 | Rupture of papillary muscle as current complication following acute myocardial infarction |
|  | I23.6 | Thrombosis of atrium, auricular appendage, and ventricle as current complication following acute myocardial infarction |
|  | I23.8 | Other current complications following acute myocardial infarction |
|  | I24.1 | Dressler syndrome |
|  | I25.2 | Old myocardial infarction |
| Angina Pectoris (both stable and unstable) | I20.0 | Unstable angina |
|  | I20.1 | Angina pectoris with documented spasm |
|  | I20.8 | Other forms of angina pectoris |
|  | I20.9 | Angina pectoris, unspecified |
| Ischemic Stroke | I63.0 | Cerebral infarction due to thrombosis of precerebral arteries |
|  | I63.1 | Cerebral infarction due to embolism of precerebral arteries |
|  | I63.3 | Cerebral infarction due to thrombosis of cerebral arteries |
|  | I63.4 | Cerebral infarction due to embolism of cerebral arteries |
|  | I63.5 | Cerebral infarction due to unspecified occlusion or stenosis of cerebral arteries |
|  | I63.6 | Cerebral infarction due to cerebral venous thrombosis, nonpyogenic |
|  | I63.8 | Other cerebral infarction |
|  | I63.9 | Cerebral infarction, unspecified |
