# Supplementary material for: Multiple metabolic comorbidities and their consequences among patients with peripheral arterial disease
Source: PLoS One. 2022 May 10;17(5):e0268201. doi: 10.1371/journal.pone.0268201 (PMC9089858; doi:10.1371/journal.pone.0268201)
Supplement: S4 Table — (DOCX) [file pone.0268201.s004.docx]

S4 Table. Health Insurance Review and Assessment Service (HIRA) Procedures codes

| Major Amputation | N0571 | Pelvis |
| --- | --- | --- |
|  | N0572 | Thigh |
|  | N0573 | Upper Arm, forearm, Lower Leg |
| Minor Amputation | N0574 | Hand, Foot |
|  | N0575 | Finger, Toe |
| Endovascular Revascularization | M6595 | Percutaneous Transluminal (balloon) angioplasty, aorta |
|  | M6597 | Percutaneous Transluminal (balloon) Angioplasty-Others |
|  | M6603 | Percutaneous Intravascular Installation of Metallic Stent-Aortic |
|  | M6605 | Percutaneous Intravascular Installation of Metallic Stent-Others |
|  | M6620 | Percutaneous Intravascular Atherectomy |
|  | M6613 | Percutaneous Intravascular Installation of Stent Graft-Others |
|  | M6632 | Percutaneous Thrombus Removal-Thrombolytic Treatment-Others |
| Open-surgery | O0161 | Vascular Bypass Operation(Femoral-Femoral, Clavicle-Clavicle Or Axilla-Axilla),Autologous Vessel |
|  | O0162 | Vascular Bypass Operation(Femoral-Femoral, Clavicle-Clavicle Or Axilla-Axilla),Artificial Vessel |
|  | O0163 | Vascular Bypass Operation(Femoral-Popliteal[Above Knee Joint]),Autologous Vessel |
|  | O0164 | Vascular Bypass Operation(Femoral-Popliteal[Knee Joint Upper]),Artificial Vessel |
|  | O0165 | Vascular Bypass Operation(Femoral-Popliteal[Below Knee Joint]),Autologous Vessel |
|  | O0166 | Vascular Bypass Operation(Femoral-Popliteal[Below Knee Joint]),Artificial Vessel |
|  | O0167 | Vascular Bypass Operation(Femoral-Tibia,Fibula),Autologous Vessel |
|  | O0168 | Vascular Bypass Operation(Femoral-Tibia,Fibula),Artificail Vessel |
|  | O0169 | Vascular Bypass Operation(Popliteal-Tibia,Fibula),Autologous Vessel |
|  | O0170 | Vascular Bypass Operation(Popliteal-Tibia,Fibula),Artificial Vessel |
|  | O0171 | Vascular Bypass Operation(Axilla-Femoral),Artificial Vessel |
|  | O0226 | Transluminal Atherectomy-Carotid Artery(Simple) |
|  | O0227 | Transluminal Atherectomy-Carotid Artery(Complex) |
|  | O1643 | Vascular Bypass Op(Aorta-Renal,Thoracic,Abdominal Aorta-Femoral,Aorta-Splanchnic),Autologous Vessel |
|  | O1644 | Vascular Bypass Op(Aorta-Renal,Thoracic,Abdominal Aorta-Femoral,Aorta-Splanchnic),Artificial Vessel |
|  | O1645 | Vascular Bypass Operation(Artery-Others),Autologous Vessel |
|  | O1646 | Vascular Bypass Operation(Artery-Others),Artificial Vessel |
|  | O1950 | Pulmonary Artery Embolectomy |
|  | O2054 | Thrombectomy(Artery),Abdomen |
|  | O2055 | Thrombectomy(Artery),Neck |
|  | O2056 | Thrombectomy(Artery),Others |
|  | O2058 | Thrombectomy(Deep Vein),Abdomen |
|  | O2059 | Thrombectomy(Deep Vein),Others |
|  | O2064 | Transluminal Atherectomy-Abdominal Artery or Iliac Artery |
|  | O2065 | Transluminal Atherectomy-Others |
|  | O2066 | Transluminal Atherectomy-Carotid Artery |
|  | O2067 | Transluminal Atherectomy-Abdominal Artery or Iliac Artery |
|  | O2068 | Transluminal Atherectomy-Others |
|  | OA632 | Angioplasty(End-to-End Anastomosis)-By Laparotomy |
|  | OA633 | Angioplasty(End-to-End Anastomosis)-Others |
|  | OA636 | Angioplasty(With Patch Graft)-By Laparotomy,Artificial Vessel |
|  | OA637 | Angioplasty(With Patch Graft)-By Laparotomy,Autologous Vessel |
|  | OA638 | Angioplasty(With Patch Graft)-Others,Artificial Vessel |
|  | OA639 | Angioplasty(With Patch Graft)-Others,Autologous Vessel |
|  | OB632 | Angioplasty(End-to-End Anastomosis)-By Laparotomy |
|  | OB633 | Angioplasty(End-to-End Anastomosis)-Others |
|  | OB636 | Angioplasty(With Patch Graft)-By Laparotomy,Artificial Vessel |
|  | OB637 | Angioplasty(With Patch Graft)-By Laparotomy,Autologous Vessel |
|  | OB638 | Angioplasty(With Patch Graft)-Others,Artificial Vessel |
|  | OB639 | Angioplasty(With Patch Graft)-Others,Autologous Vessel |
