# Supplementary material for: Multiple metabolic comorbidities and their consequences among patients with peripheral arterial disease
Source: PLoS One. 2022 May 10;17(5):e0268201. doi: 10.1371/journal.pone.0268201 (PMC9089858; doi:10.1371/journal.pone.0268201)
Supplement: S5 Table — A. Anti-hypertensive medications. B. Anti-diabetic medications. C. Anti-dyslipidemic medications. D. Antiplatelet medications. (DOCX) [file pone.0268201.s005.docx]

S5A Table. Anti-hypertensive medications

|  | Ingredient |
| --- | --- |
| Thiazide diuretics & thiazide-likes diuretics | hydrochlorothiazide |
|  | chlothalidone |
|  | indapamide |
|  | metolazone |
|  | tripamide |
|  | xipamide |
| Loop diuretics | azosemide |
|  | furosemide |
|  | torsemide |
| K+ sparing diuretics | amiloride |
|  | spironolactone |
| Central alpha 2 agonists | clonidine |
|  | methyldopa |
|  | moxonidine |
| Alpha blockers | doxazosin |
|  | prazosin |
|  | terazosin |
|  | urapidil |
|  | phenosybenzamine |
| Beta blockers | s-atenolol |
|  | acebutolol |
|  | amosulalol |
|  | arotinolol |
|  | atenolol |
|  | betaxolol |
|  | bevantolol |
|  | bisoprolol |
|  | carteolol |
|  | carvedilol |
|  | celiprolol |
|  | metoprolol |
|  | propranolol |
|  | labetalol |
|  | nadolol |
|  | nebivolol |
|  | penbutolol |
| Vasodilators | cadralazine |
|  | hydralazine |
|  | minoxidil |
| Calcium channel blockers (DHP) | amlodipine |
|  | lercanidipine |
|  | nifedipine |
|  | nisoldipine |
|  | nitrendifine |
|  | barnidipine |
|  | benidipine |
|  | cilnidipine |
|  | efonidipine |
|  | Felodipine |
|  | isradipine |
|  | lacidipine |
|  | manidipine |
|  | nicardipine |
|  | nilvadipine |
|  | s-ampodipine |
| Calcium channel blockers (non-DHP) | verapamil |
|  | diltiazem |
| Angiotensin Converting Enzyme inhibitors | alacepril |
|  | benazepril |
|  | captopril |
|  | cilazapril |
|  | delapril |
|  | enalapril |
|  | fosinopril |
|  | imidapril |
|  | lisinopril |
|  | moexipril |
|  | perindopril |
|  | quinapril |
|  | Ramipril |
|  | spirapril |
|  | temocapril |
|  | trandolapril |
| Angiotensin receptor blockers | candesartan |
|  | eposartan |
|  | irbesartan |
|  | losartan |
|  | olmesartan |
|  | telmisartan |
|  | valsartan |
| Other hypertensive medications | amlodipine+atrovastatin |
|  | amlodipine+fimasartan |
|  | amlodipine+losartan |
|  | amlodipine+olmesartan |
|  | amlodipine+olmesarten |
|  | amlodipine+rosuvastatin |
|  | amlodipine+telmisartan |
|  | amlodipine+telmisartan |
|  | amlodipine+valsartan |
|  | atenolol+chlorhalidone |
|  | atorvastatin+irbesartan |
|  | bisoprolol+hydrochlorothiazide |
|  | candersartan+hydrochlorothiazide |
|  | captopril+hydrochlorothiazide |

S5B Table. Anti-diabetic medications

|  | Ingredient |
| --- | --- |
| Single medicine | acarbose |
|  | glibenclamide |
|  | gliclazide |
|  | glipizide |
|  | glimepiride |
|  | metformin hydrochloride |
|  | voglibose |
|  | rosiglitazone maleate |
|  | repaglinide |
|  | miglitol |
|  | meglitinides |
|  | nateglinide |
|  | pioglitazone hydrochloride |
|  | mitiglinide calcium hydrate |
|  | vildagliptin |
|  | sitagliptin phosphate hydrate |
|  | lobeglitazone sulfate |
|  | dapagliflozin propanediol hydrate |
|  | saxagliptin hydrate |
|  | linagliptin |
|  | gemigliptin tartrate sesquihydrate |
|  | alogliptin benzoate |
|  | teneligliptin hydrobromide hydrate |
|  | empagliflozin |
|  | ipragliflozin L-proline |
|  | anagliptin |
|  | evogliptin tartrate |
| Combination medicine | glibenclamide + metformin hydrochloride |
|  | rosiglitazone maleate + metformin hydrochloride |
|  | gliclazide + metformin hydrochloride |
|  | glimepiride + metformin hydrochloride |
|  | pioglitazone hydrochloride + metformin hydrochloride |
|  | sitagliptin phosphate hydrate + metformin hydrochloride |
|  | vildagliptin+metformin hydrochloride |
|  | saxagliptin hydrate + metformin hydrochloride |
|  | mitiglinide calcium hydrate + metformin hydrochloride |
|  | linagliptin + metformin hydrochloride |
|  | voglibose + metformin hydrochloride |
|  | gemigliptin tartrate sesquihydrate + metformin hydrochloride |
|  | repaglinide + metformin hydrochloride |
|  | alogliptin benzoate + metformin hydrochloride |
|  | teneligliptin hydrobromide hydrate + Metformin hydrochloride |
|  | glimepiride + rosiglitazone maleate |
|  | glimepiride + pioglitazone hydrochloride |
|  | alogliptin benzoate + pioglitazone hydrochloride |

S5C Table. Anti-dyslipidemic medications

|  | Category | Ingredient |
| --- | --- | --- |
| single medicine | statins | atorvastatin |
|  |  | fluvastatin |
|  |  | lovastatin |
|  |  | pitavastatin |
|  |  | pravastatin |
|  |  | rosuvastatin |
|  |  | simvastatin |
|  | fibrates | bezafibrate |
|  |  | ciprofibrate |
|  |  | etofibrate |
|  |  | fenofibrate |
|  |  | gemifibrozil |
|  | BAR (Bile acid sequestrants) | cholestyramine |
|  | niacin | acipimox |
|  |  | nicotinic acid |
|  | omega-3 | omega-3-acid ethyl esters90 |
|  | ezetimibe | ezetimibe |
| combination medicine |  | ezetimibe+simvastatin |
|  |  | ezetimibe+atrovastatin |
|  |  | ezetimibe+rosuvastatin |
|  |  | Pravastatin+fenofibrate |
|  |  | simvastatin+fenofibrate |

S5D Table. Antiplatelet medications

|  | Ingredient |
| --- | --- |
| single medicine | aspirin |
|  | clopidogrel |
|  | ticlodipine |
|  | Ticagrelor |
|  | Prasugrel |
|  | Sarpogrelate |
|  | Triflusal |
|  | Beraprost |
|  | Limaprost |
|  | Warfarin |
|  | Cilostazol |
| combination medicine | aspirin+dipyridamole |
|  | aspirin+clopidogrel |
